# Proxy gene-by-environment Mendelian randomization study confirms a causal effect of maternal smoking on offspring birthweight, but little evidence of long-term influences on offspring health

**DOI:** 10.1093/ije/dyz250

**Published:** 2019-12-13

**Authors:** Qian Yang, Louise A C Millard, George Davey Smith

**Affiliations:** 1 Medical Research Council Integrative Epidemiology Unit, University of Bristol, Bristol, UK; 2 Population Health Sciences, University of Bristol, Bristol, UK; 3 Intelligent Systems Laboratory, Department of Computer Science, University of Bristol, Bristol, UK

**Keywords:** Gene ×, environment, Mendelian randomization, proxy, maternal smoking, pregnancy

## Abstract

**Background:**

A lack of genetic data across generations makes transgenerational Mendelian randomization (MR) difficult. We used UK Biobank and a novel proxy gene-by-environment MR to investigate effects of maternal smoking heaviness in pregnancy on offspring health, using participants’ (generation one: G1) genotype (rs16969968 in *CHRNA5*) as a proxy for their mothers’ (G0) genotype.

**Methods:**

We validated this approach by replicating an established effect of maternal smoking heaviness on offspring birthweight. Then we applied this approach to explore effects of maternal (G0) smoking heaviness on offspring (G1) later life outcomes and on birthweight of G1 women’s children (G2).

**Results:**

Each additional smoking-increasing allele in offspring (G1) was associated with a 0.018 [95% confidence interval (CI): -0.026, -0.009] kg lower G1 birthweight in maternal (G0) smoking stratum, but no meaningful effect (-0.002 kg; 95% CI: -0.008, 0.003) in maternal non-smoking stratum (interaction *P*-value = 0.004). The differences in associations of rs16969968 with grandchild’s (G2) birthweight between grandmothers (G0) who did, versus did not, smoke were heterogeneous (interaction *P*-value = 0.042) among mothers (G1) who did (-0.020 kg/allele; 95% CI: -0.044, 0.003), versus did not (0.007 kg/allele; 95% CI: -0.005, 0.020), smoke in pregnancy.

**Conclusions:**

Our study demonstrated how offspring genotype can be used to proxy for the mother’s genotype in gene-by-environment MR. We confirmed the causal effect of maternal (G0) smoking on offspring (G1) birthweight, but found little evidence of an effect on G1 longer-term health outcomes. For grandchild’s (G2) birthweight, the effect of grandmother’s (G0) smoking heaviness in pregnancy may be modulated by maternal (G1) smoking status in pregnancy.

**Key Messages**

- Our study presents a novel proxy gene-by-environment (G × E) Mendelian randomization (MR) approach to explore maternal effects on offspring phenotypes when maternal genetic information is unavailable.
- We demonstrated the proxy G × E MR approach in UK Biobank, showing that heavier maternal smoking led to lower offspring birthweight, which has been demonstrated using several study designs.
- Using this method, we found little evidence that maternal smoking heaviness in pregnancy was associated with offspring’s height, body mass index, lung function, risk of asthma, blood pressure, age at menarche, years of education, intelligence score, depression/anxiety or happiness in adulthood.
- Our study also suggested that the effect of grandmother’s smoking heaviness in pregnancy on grandchild’s birthweight may be modulated by maternal smoking status in pregnancy.
- Further G × E MR studies with larger sample sizes are needed to assess transgenerational effects with greater statistical power.

## Introduction

The developmental origins of health and disease hypothesis proposes that early life experiences, including those *in utero*, can have long-term health effects, and maternal pregnancy exposures are important to long-term health of offspring.^1^ Heavier maternal smoking in pregnancy is known to be causally associated with lower offspring birthweight,^2–6^ but its other effects in offspring are less clear. Multivariable regression in observational data showed that heavier maternal smoking during pregnancy was associated with offspring being shorter^7^ and more overweight/obese,^8^^,^^9^ and having higher blood pressure,^10^ but had mixed associations with age at menarche^11^ and respiratory,^12^ cognitive^13^ and mental health.^14^ Heavier maternal smoking in pregnancy has also been associated with higher grandchild’s birthweight in certain subpopulations.^15–17^ It is unclear whether these associations reflect a causal effect of maternal smoking in pregnancy, as they may be due to residual confounding. Some studies have assessed this using paternal smoking as a ‘negative control’, since an effect via uterine environment would be observed in mothers but not fathers, such that similar-magnitude associations would indicate confounding via shared familial, social, environmental and genetic factors.^2^^,^^5^^,^^18^ Negative control studies suggest little evidence of a causal effect on offspring body mass index (BMI),^2^^,^^5^^,^^8^ blood pressure^19^^,^^20^ and depression.^21^

Mendelian randomization (MR) provides an alternative way to explore this question by using single nucleotide polymorphisms (SNPs) as instrumental variables (IVs) for an exposure of interest. MR is less prone to confounding, as germline genetic variants are randomly allocated at meiosis and are not influenced by subsequent socioeconomic and health behaviours.^22^^,^^23^ MR has been applied in a gene-by-environment (G × E) framework,^24^^,^^25^ which requires variation in the strength of the gene-exposure association across strata of another factor. If there is a causal effect of the IV on the outcome via the exposure of interest, then we would expect the association of the IV with the outcome to vary in proportion to the gene-exposure association. The rs1051730/rs16969968 (*CHRNA5*) SNPs, previously robustly associated with smoking heaviness among smokers,^26^ have been widely used as IVs for smoking heaviness in G× E MR studies.^3^^,^^27–29^ A causal effect of the smoking heaviness IV on an outcome should be seen among ever but not among never smokers if the effect is via smoking heaviness rather than other pathways.^24^^,^^25^ G × E MR has also been used to assess cross-generational causal effects. A smoking heaviness IV has been associated with lower offspring birthweight among mothers who smoked in pregnancy but not among mothers who did not smoke in pregnancy, suggesting that the genetic instrument affects birthweight through maternal smoking.^3^

It is usually difficult to investigate transgenerational associations, due to a lack of data across the generations of interest. Thus, previous work has sought to test transgenerational associations using available traits as proxies for unmeasured traits of interest. A Norwegian cohort aimed to examine whether women’s smoking in adulthood was related to their mothers’ smoking habits (that were not recorded) and hence used maternal smoking-related mortality as a proxy.^30^ Recently, a case-control by proxy approach has been proposed.^31^ Participants’ genotypes were used to proxy for unavailable parental genotypes, and their associations were tested against parental diagnosis of Alzheimer’s disease in UK Biobank,^31^ since Alzheimer’s disease was much more prevalent in the parents than in the participants (aged between 40 and 69 at baseline in 2006–10^32^). Our study aimed to demonstrate how an analogous approach can be used within a G × E MR framework to test maternal-offspring effects when maternal genotype is not available, using offspring genotype as a proxy for the maternal genotype. First, we performed a proof of principle analysis to demonstrate this approach, testing the previously established finding that maternal smoking in pregnancy leads to lower offspring birthweight. Second, we tested for causal effects of maternal smoking on offspring later life outcomes. Finally, we tested for a causal effect of grandmother’s smoking on grandchild’s birthweight.

## Methods

### Study population

Our study was conducted using UK Biobank, a population-based cohort of more than 500 000 men and women in the UK. The UK Biobank received ethical approval from the research ethics committee (REC reference for UK Biobank 11/NW/0382) and participants provided written informed consent.

This study collected a large and diverse range of data from physical measures, questionnaires and hospital episode statistics.^32^ Of 463 013 participants of European descent with genetic data passing initial quality control (i.e. genetic sex same as reported sex, XX or XY in sex chromosome and no outliers in heterozygosity and missing rates),^33^ 289 684 participants (54% women) of White British descent were eligible for inclusion in our analyses (Supplementary Figure 1, available as Supplementary data at *IJE* online). We refer to the UK Biobank participants as generation one (G1), and their mothers and offspring as G0 and G2, respectively.

### Genetic IV for maternal (G0) smoking

The rs16969968 SNP located in *CHRNA5* has been robustly associated with smoking heaviness.^26^ Ideally, we would use the maternal rs16969968 as an IV for the heaviness of maternal smoking, but in UK Biobank parental genetic data are not available. Hence, we used rs16969968 of the UK Biobank participants (G1) as a proxy for that of their mothers, coded as the number of smoking heaviness-increasing alleles.

### Smoking phenotypes

We used participants’ answers to the question ‘Did your mother smoke regularly around the time when you were born?’ as a proxy for G0 smoking during pregnancy. Participants were also asked to report their smoking status (current/former/never). We derived a binary ever versus never measure of smoking status by combining current and former smokers. We derived a measure denoting whether G1 women who had at least one live birth smoked during the pregnancy of their first child. G1 women who gave birth to their first child at age b were assigned as non-smokers in pregnancy if they: (i) never smoked; (ii) currently smoked but started smoking when b + 1 years old or later; (iii) formerly smoked but started smoking when b + 1 years old or later; or (iv) formerly smoked but stopped smoking when b-2 years old or earlier. Smokers included: (i) current smokers who started smoking when b-1 years old or earlier; and (ii) former smokers who started smoking when b-1 years old or earlier but stopped smoking when b + 1 years old or later. As these ages were recorded as whole numbers of years, it was not always possible to determine whether a woman was a smoker in pregnancy (illustrated in Supplementary Figure 2, available as Supplementary data at *IJE* online).

### Outcomes in participants (G1)

We used baseline data measured at the UK Biobank initial assessment centre. Anthropometric traits included participants’ birthweight (kg, self-reported), standing height (cm) and BMI (kg/m^2^, constructed from standing height and weight). To assess lung function, forced vital capacity (L) and forced expiratory volume in 1 s (L) were measured by spirometry. Participants reported whether they had had asthma via the question ‘Has a doctor ever told you that you have had any of the following conditions?’ (with an option of asthma).^34^ Systolic and diastolic blood pressure (mmHg) were measured twice using a digital monitor, or a manual sphygmomanometer if the digital monitor could not be employed, and we took the average of the two readings. Female participants reported their age at menarche. We derived years of education based on qualifications achieved by participants, as described previously.^35^ We included follow-up data of a subset of participants to define intelligence and depression/anxiety. Fluid intelligence score was generated as an unweighted sum of the number of correct answers given to 13 questions, and we used the earliest score if we had data at multiple time points.^36^ We defined depression/anxiety cases as participants that either answered ‘Yes’ to ‘Have you ever seen a general practitioner (GP) for nerves, anxiety, tension or depression?’ or to ‘Have you ever seen a psychiatrist for nerves, anxiety, tension or depression?’, or had hospital episode coded using ICD-10.^37^ Happiness was assessed via a question – ‘In general how happy are you?’, with six categories ranging from ‘extremely happy’ to ‘extremely unhappy’.

### Outcomes in participants’ offspring (G2)

The female participants with at least one live birth were asked to report their first child’s birthweight. Male participants were not asked to report the birthweight of their offspring.

### Statistical analyses

Proof of principle analysis implies testing the causal effect of maternal (G0) smoking heaviness in pregnancy on participants’ (G1) birthweight

**Figure 1:**
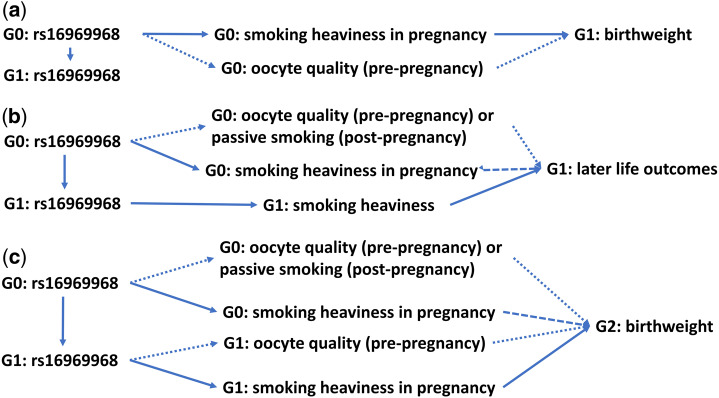
Directed acyclic graphs (DAGs) of this study. Generation (G)0: UK Biobank participants’ mothers; G1: UK Biobank participants themselves; G2: first offspring of UK Biobank participants. (a) Assessing the effect of G0 smoking heaviness on G1 birthweight: We used G1 rs16969968 as a proxy for G0 rs16969968 and stratified on G0 smoking status in pregnancy. G1 rs16969968 could have no effect on G1 birthweight via G1 smoking heaviness, since G1 cannot smoke before they were born. Maternal smoking outside pregnancy might influence the outcome, e.g. via oocyte quality, causing an alternative path between rs16969968 and G1 birthweight (shown as dotted →) (b) Assessing the effect of G0 smoking on G1 later life outcomes: Besides the paths described in (a), G1 rs16969968 could influence the outcomes via G1 smoking heaviness. To estimate the effect of G0 smoking heaviness in pregnancy (shown as dashed →), we need to block this by further stratifying on G1 smoking status. (c) Assessing the effect of G0 smoking on G2 birthweight: besides the paths described in (a), G1 rs16969968 could influence G2 birthweight via G1 smoking heaviness in pregnancy. To estimate the effect of G0 smoking heaviness in pregnancy (shown as dashed →), we need to block this by further stratifying on G1 smoking status in pregnancy. G1 pre-pregnancy smoking might influence G2 birthweight (shown as dotted →). See further DAGs in Supplementary Figure 3 (available as Supplementary data at *IJE* online) illustrating potential sources of bias due to conditioning on a collider.

In this proof of principle analysis, we seek to replicate the finding, previously established using G × E MR and many other methods,^6^ that heavier maternal smoking causes lower offspring birthweight. We use our proxy G× E approach, where participants’ (G1) genotype is used as a proxy for their mothers’ (G0) genotype. To assess whether G1 rs16969968 affects G1 birthweight via G0 smoking in pregnancy, we stratified our G1 sample by G0 smoking status during pregnancy, and then tested the associations of rs16969968 with birthweight in each stratum using multivariable linear regression. Since birth precedes smoking initiation, participants’ genotype cannot affect birthweight through their own smoking heaviness, which means we do not need to consider smoking status of participants (Figure 1a). We included participants’ sex as a covariate to reduce variation in their birthweight, and the first 10 principal components to control for population stratification. We assumed an additive genetic effect and identified the strength of interaction between strata using Cochran’s Q test for heterogeneity.

### Testing for causal effects of G0 smoking in pregnancy on G1 later life outcomes

We use the proxy G× E MR approach to test for causal effects of maternal (G0) smoking heaviness on offspring (G1) height, BMI, lung function, asthma, blood pressure, age at menarche, education, intelligence, depression/anxiety and happiness. In contrast to our proof of principle example where participants (G1) smoking in adulthood cannot influence their birthweight, participants’ rs16969968 could affect these outcomes via both maternal (G0) and participants’ (G1) smoking heaviness (Figure 1b). To assess whether rs16969968 may affect these outcomes via maternal versus participants’ smoking, we stratified on both maternal and participants’ smoking status. In each stratum, we examined associations of rs16969968 with height, BMI, lung function, blood pressure, age at menarche, education and intelligence using linear regression, with asthma and depression/anxiety using logistic regression and with happiness using ordinal logistic regression. We included participants’ age at baseline, sex and the first 10 genetic principal components as covariates.

Height and age at menarche manifest around the time of puberty, such that participants’ own smoking can only affect these if they started smoking before these outcomes are determined. We conducted sensitivity analyses for these outcomes stratifying G1 participants according to whether they were ever smokers before achieving their adulthood height (assuming age at 17 for men and 15 for women^38^) or their age at menarche.

### Testing for causal effects of G0 smoking in pregnancy on grandchild’s (G2) birthweight

To test for a causal effect of participants’ mothers’ (G0) smoking on birthweight of participants’ offspring (G2), we stratified G1 women based on their own and their mothers’ smoking status during pregnancy, as rs16969968 could affect G2 birthweight through both G0 and G1 smoking heaviness (Figure 1c). Within each stratum, we assessed associations of G1 rs16969968 with G2 birthweight using linear regression, adjusting for the first 10 genetic principal components. We estimated the strength of interaction between G0 smokers and G0 non-smokers within each G1 stratum. We also calculated a difference^39^ in those associations between G0 smokers and G0 non-smokers within each G1 stratum, and estimated the strength of interaction between two differences to investigate whether G1 smoking status modulates the effect of G1 rs16969968 on G2 birthweight.

Our G × E MR may be vulnerable to collider bias^29^^,^^40^^,^^41^ (see details in Discussion), so we tested associations of G1 rs16969968 with G0 and G1 smoking status and potential confounders available in UK Biobank. We performed simulations to compare statistical power of proxy G × E MR with that of G × E MR (see Supplementary Methods, available as Supplementary data at *IJE* online). We also tested observational associations of maternal (G0) smoking status with offspring (G1) smoking status and all outcomes for comparison with our MR results. Analyses were performed using R version 3.5.1 (R Foundation for Statistical Computing, Vienna). All code used to produce the results can be accessed at [https://github.com/MRCIEU/MR-maternal-smoking]. Git tag v0.2 corresponds to the version presented here.

## Results

Characteristics of participants stratified by sex are shown in Supplementary Table 1 (available as Supplementary data at *IJE* online). Each additional smoking-increasing allele of participants’ (G1) rs16969968 was associated with a 1.02 [95% confidence interval (CI): 1.01, 1.03; *P*-value = 5 × 10^–3^] higher odds of their mothers’ (G0) smoking in pregnancy, a 0.98 (95% CI: 0.97, 0.99; *P*-value = 7 × 10^–4^) lower odds of being an ever (versus never) smoker themselves, and a 1.06 (95% CI: 1.04, 1.09; *P*-value = 3 × 10^–7^) higher odds that female participants were smokers (versus non-smokers) in their own pregnancy. We found little evidence of an association between rs16969968 and potential confounders, with small associations for participants’ age and years of education in some strata (Supplementary Table 2, available as Supplementary data at *IJE* online).

**Figure 2:**
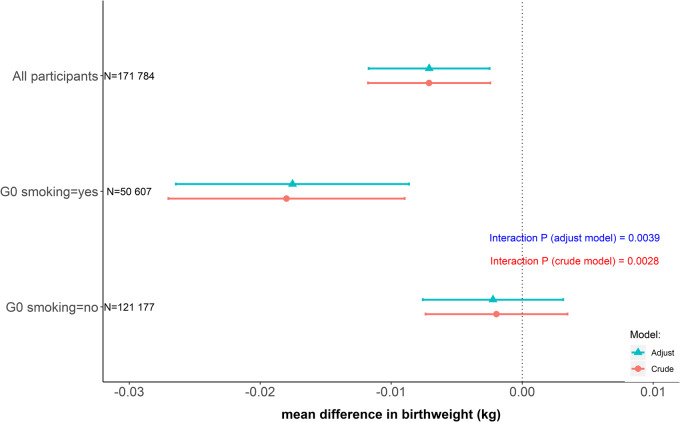
The associations of rs16969968 of UK Biobank participants with their own birthweight by their mothers’ smoking status during pregnancy. Generation (G)0: UK Biobank participants’ mothers; G1: UK Biobank participants themselves. Estimates are the mean difference of G1 birthweight per each smoking heaviness-increasing allele of rs16969968. In the crude model, we did not adjust for covariates; in the adjusted model, we adjusted for sex of participants and the first 10 genetic principal components. The number of participants was listed for each analysis.

Our proof of principle analysis found that, among participants (G1) whose mothers (G0) smoked in pregnancy, each additional smoking-increasing allele in G1 was associated with a 0.018-kg lower G1 birthweight (95% CI: -0.026, -0.009) after adjustment for covariates (Figure 2). Among participants whose mothers did not smoke in pregnancy, we found little evidence for an association of rs16969968 with birthweight [-0.002 kg (95% CI: -0.008, 0.003)], and we observed heterogeneity between these associations (interaction *P*-value = 0.004). Estimates were consistent before and after adjustment for covariates.

Figure 3 shows estimates of G1 rs16969968 on the 12 outcomes in the UK Biobank participants (G1). Overall, within each stratum, the estimates were broadly consistent between those whose mothers smoked and those whose mothers did not, except for height among participants who never smoked (all interaction *P*-values are in Supplementary Table 3, available as Supplementary data at *IJE* online). Each additional smoking-increasing allele was associated with a 0.115-cm lower height (95% CI: -0.200, -0.030) among G1 never smokers whose mothers smoked in pregnancy, but a 0.002-cm lower height (95% CI: -0.057, 0.053) among G1 never smokers whose mothers did not smoke in pregnancy (interaction *P*-value = 0.029). However, this difference was not observed among G1 ever smokers (Figure 3a). We obtained largely consistent results in sensitivity analyses (Supplementary Figure 3, available as Supplementary data at *IJE* online).

**Figure 3:**
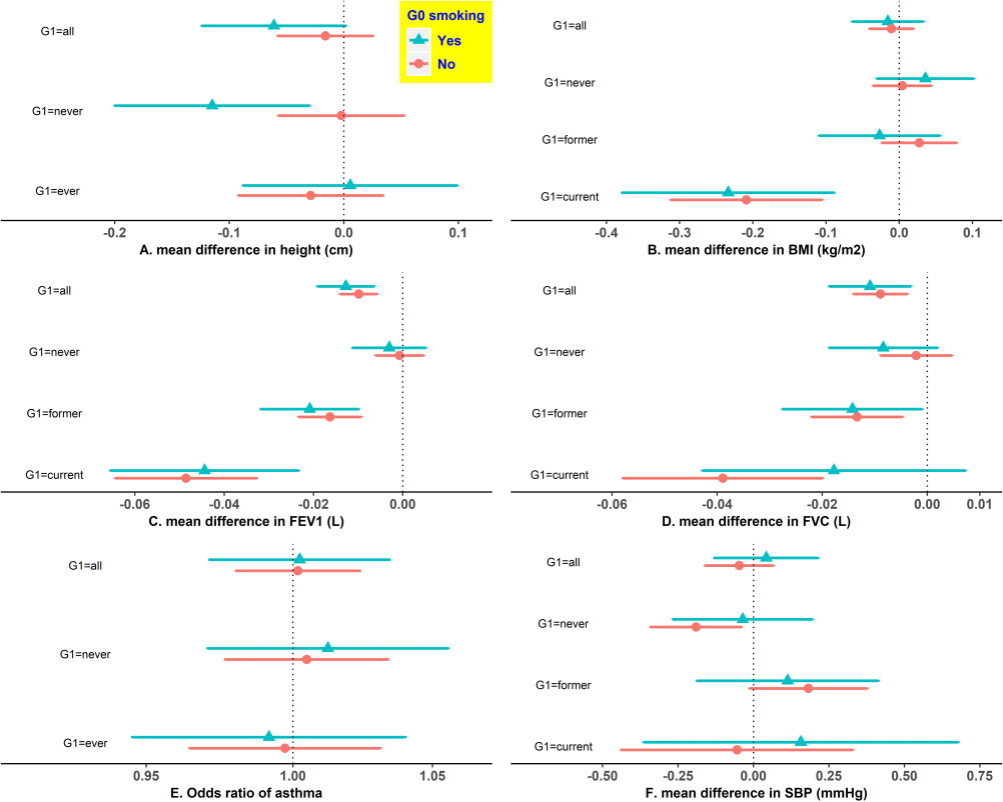
The associations of participants’ rs16969968 with 12 outcomes in UK Biobank participants by their mothers’ smoking status during pregnancy and their own smoking status. Generation (G)0: UK Biobank participants’ mothers; G1: UK Biobank participants themselves. Estimates are the mean difference (or change in odds) of G1 outcome per each smoking heaviness-increasing allele of rs16969968. We adjusted for age and sex of participants for outcomes except for menarche, and the first 10 principal components for all 12 outcomes. We combined G1 current and former smokers into ever smokers for height, menarche, education, asthma and happiness, to enlarge sample sizes given smoking cessation may not have a rapid impact on them. BMI, body mass index; DBP, diastolic blood pressure; FEV1, forced expiratory volume in 1 s; FVC, forced vital capacity; SBP, systolic blood pressure.

**Figure 3:**
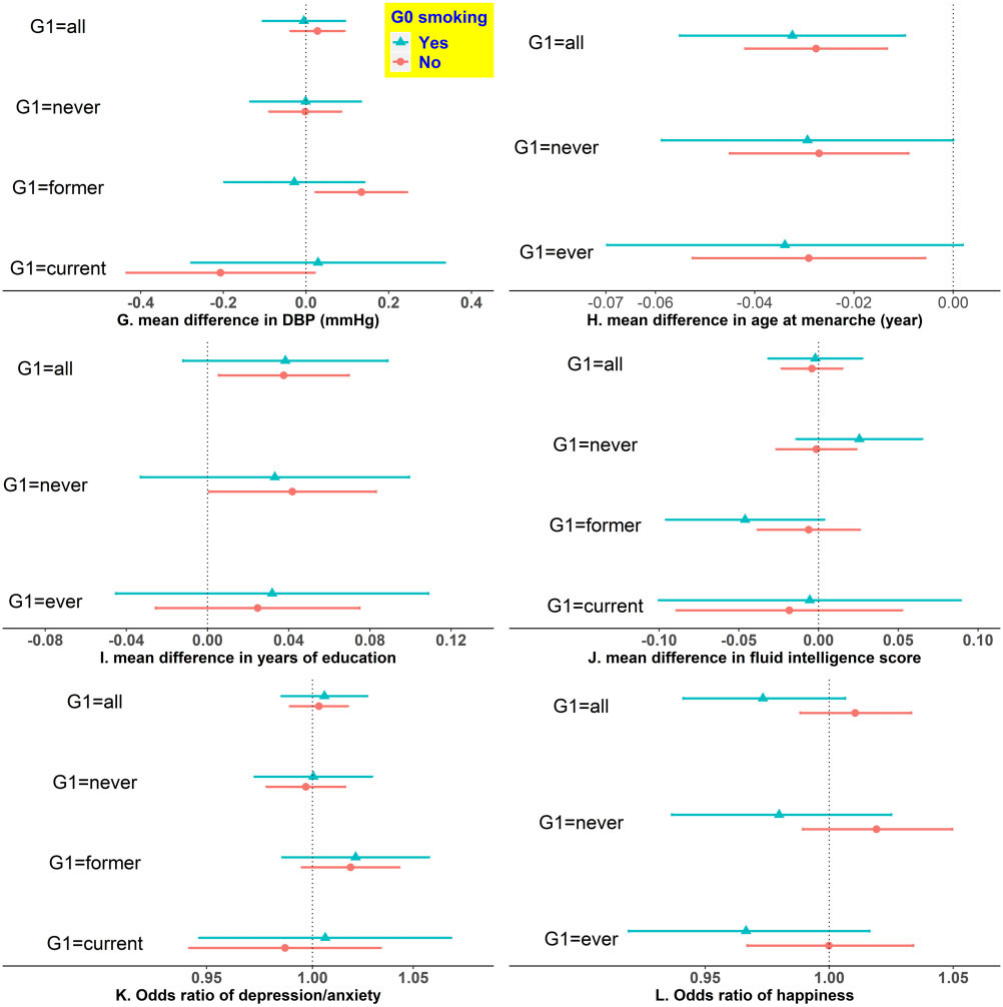
Continued.

**Figure 4:**
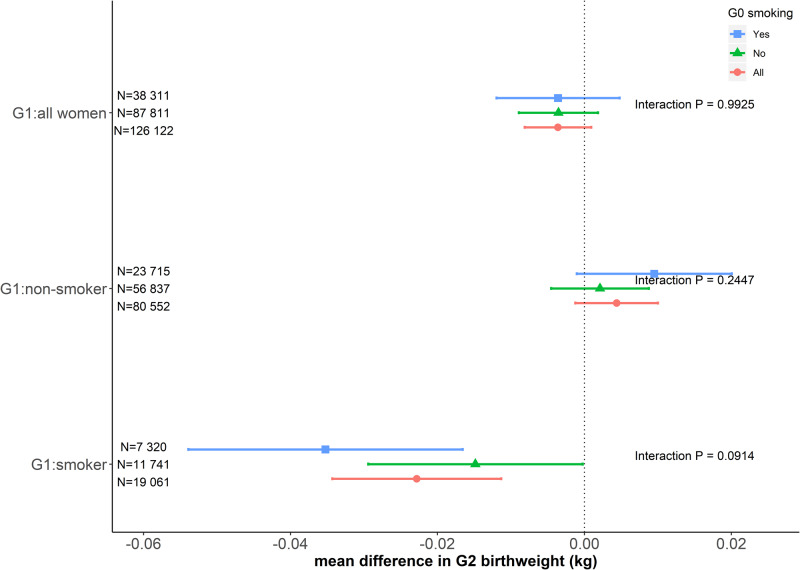
The associations of rs16969968 of UK Biobank women participants with their first child’s birthweight by their mothers’ and their own smoking status during pregnancy, after adjusting for the first 10 genetic principal components. Generation (G)0: UK Biobank participants’ mothers; G1: UK Biobank participants themselves; G2: first offspring of UK Biobank participants. Estimates are the mean difference of G2 birthweight per each smoking heaviness-increasing allele of rs16969968. Interactions are tested between G0 smokers (blue line) and non-smokers (green line) with their *P*-values presented. All women in G1 included G1 smokers, G1 non-smokers and G1 women whose smoking status in pregnancy was missing.

Figure 4 shows results of our analysis testing the effect of grandmother (G0) smoking heaviness on grandchild’s (G2) birthweight. Among mothers (G1) who did not smoke in pregnancy, the association of G1 rs16969968 with grandchild’s birthweight in the stratum of grandmother smoking in pregnancy was 0.007 kg/allele (95% CI: -0.005, 0.020) higher than that in the stratum of grandmother not smoking in pregnancy. However, among mothers who smoked in pregnancy, this difference was -0.020 kg/allele (95% CI: -0.044, 0.003). These two differences [pertaining to maternal (G1) smoking strata] were heterogeneous [-0.028 kg per allele (95% CI: -0.055, -0.001); interaction *P*-value = 0.042).

The directions of observational estimates were consistent with our MR estimates for both participants’ (G1) and their children’s (G2) birthweight. Our observational analyses also found associations of maternal (G0) smoking in pregnancy with offspring (G1) later life outcomes, where smoking in pregnancy was associated with lower height, higher BMI, poorer lung function, higher risk of asthma, earlier age at menarche, higher blood pressure and poorer cognitive and mental health (Supplementary Table 4, available as Supplementary data at *IJE* online).

## Discussion

In this study, we have demonstrated how G × E MR can be used to test transgenerational causal effects of maternal (G0) smoking heaviness in pregnancy, using offspring (G1) genotype as a proxy for G0 genotype. Our proof of principle analysis identified an effect of heavier maternal smoking on lower offspring birthweight, consistent with previous studies.^2–6^ Our MR study also confirmed previously established causal effects of G1 smoking on their own health, where heavier smoking reduced BMI^27^ and lead to impaired lung function,^42^ but found little evidence of an effect on asthma risk^43^ or blood pressure.^28^

Our tests of effects of maternal (G0) smoking heaviness on offspring (G1) later life health outcomes were not conclusive, given a lack of precision for many of our MR estimates. We found little evidence of an effect on BMI, lung function, asthma, blood pressure, cognition, depression/anxiety or happiness. These findings were consistent with negative control studies for BMI,^2^^,^^8^ blood pressure^19^^,^^20^ and depression/anxiety,^21^ although our estimation of interactions is not directly quantitatively comparable to their estimation of effects of ever/never smoking or smoking heaviness categories in observational studies. Our MR results found little evidence to support findings from our own and previous observational studies indicating that maternal smoking led to poorer lung function,^44^ higher risk of asthma^45^^,^^46^ and lower happiness in offspring.^47^ This may be due to residual confounding in observational associations, or because of low statistical power in MR. Previous studies did not use the same cognition measurement approaches as used in UK Biobank, making our results for this outcome less comparable. We observed lower offspring adulthood height according to maternal smoking in G1 never smokers but not in G1 ever smokers, which could be a chance finding given that we tested multiple outcomes.

We found little evidence of an effect of maternal (G0) smoking in pregnancy on offspring (G1) age at menarche. However, we did find an effect of G1 rs16969968 on age at menarche across strata of both G0 and G1 smoking status, suggesting that rs16969968 may have horizontal pleiotropic effects on age at menarche (e.g. via smoking outside pregnancy). Future MR studies could examine this.^25^

Our observational results were consistent with previous observational studies^15–17^ by showing a positive association of grandmother’s (G0) smoking in pregnancy with grandchild’s (G2) birthweight after adjusting for mother’s (G1) smoking in pregnancy. Although our G × E MR was vulnerable to insufficient statistical power, we did find evidence that female G1 smoking in pregnancy modulates the effect of G0 smoking heaviness in pregnancy on G2 birthweight, consistent with previous observational findings.^15–17^ These results highlight the importance of both grandmother’s and maternal smoking in pregnancy for fetal growth, which could have implications for public health interventions aiming to reduce the prevalence of low birthweight. Two biological mechanisms have been previously suggested to explain the increased growth of offspring (G2) whose grandmothers (G0) did, versus did not, smoke, with non-smoking mothers (G1).^48^ The first theory suggests that mothers (G1) exposed as fetuses to intrauterine smoking [due to grandmother (G0) smoking in pregnancy] would biologically anticipate growth restriction in their child (G2) and thus programme them to grow faster as a pre-adaptation to the anticipated environment.^48^ The second theory suggests that offspring fetal growth may be accelerated by paternal factors, with this normally being constrained by maternal influences.^49^ However, mothers exposed as fetuses to intrauterine smoking could have impaired ability to constrain the paternal impact, creating an imbalance that would cause her child to grow faster.^48^ However, these theories cannot explain the modulation we observed, of grandmother smoking in pregnancy on grandchild’s birthweight, among mothers who did, versus did not, smoke.

We now discuss some limitations of this work. First, our proxy G × E MR used offspring genotype as a proxy for maternal genotype, and offspring rs16969968 contains 50% information from fathers. This may reduce our statistical power to detect interactions between smoking status strata. We performed simulations to assess the impact of using offspring genotype as a proxy for parental genotype, and found that power was particularly reduced in smaller samples and/or when there were smaller effects of maternal smoking on the outcome (see Supplementary Figure 5, available as Supplementary data at *IJE* online). Second, our study might be vulnerable to collider bias,^29^^,^^40^^,^^41^ as we stratified on G0/G1 smoking status (see Supplementary Figure 3, available as Supplementary data at *IJE* online). In general, collider bias occurs when a test for the total effect of an instrument (Z) on an outcome (Y) controls for a third variable (C) or a descendant of C through stratification/restriction/adjustment, opening up a non-causal pathway between Z and Y. Additionally, we used an overall sample (UK Biobank) with evidence of selection based on smoking,^50^ and we had some missing data in outcomes which may not have been at random. These may also make our MR estimates vulnerable to collider bias induced by sampling, as described by Hughes *et al*.^51^ However, previous simulations^29^^,^^52^ and our genetic associations with measured confounders indicated that collider bias may not be large enough to have a meaningful impact on our MR estimates. Third, rs16969968 predicts life course smoking heaviness and not just in pregnancy. Women who smoked in pregnancy will also smoke outside pregnancy. Therefore, the effect of maternal smoking might be via other pathways such as poor oocyte quality (influencing offspring birthweight) or postnatal maternal smoking (e.g. passive smoke exposure) influencing adulthood outcomes among offspring.^53^ Using paternal smoking in pregnancy as a negative control^2^ could further validate proxy G × E MR findings and help to separate intrauterine effects from other effects of parental smoking on offspring outcomes. Unfortunately, such paternal smoking data were not available in UK Biobank.

Fourth, both participants’ (G1) and their mothers’ (G0) smoking status may be misclassified. Participants were asked to report whether their mother smoked around the time of their birth, and we used this as our measure of G0 smoking in pregnancy. This means that G0 individuals assigned as smokers might have smoked for the duration of their pregnancy or part of their pregnancy, or they might have paused for their pregnancy and restarted smoking shortly after giving birth; whereas G0 individuals assigned as non-smokers might have smoked for a large duration of their pregnancy and stopped sometime before the time of birth. Effects of smoking heaviness in pregnancy may vary according to the duration and pregnancy period during which a woman smoked. For instance, previous work found that smoking in the first trimester was not associated with lower birthweight in offspring, suggesting that later stages may be more important for fetal growth.^3^^,^^15^ Similarly, participants reported their smoking status at baseline, but this may not reflect their smoking status at an important time point for a given outcome. For instance, participants’ height and age at menarche can only be affected by their own smoking behaviour if they started smoking before achieving adult height or the onset of puberty. We performed sensitivity analyses for height and age at menarche, using estimates of participants’ smoking status before these outcomes. For height, this assumed that men and women achieved their adult height at 17 and 15 years old,^38^ respectively, as this information was not available in UK Biobank. Fifth, we tested several hypotheses, which increases the probability that our identified associations may be due to chance. Finally, our study may lack statistical power due to small sample sizes in strata and the low power of tests for interactions.^54^ We were unable to account for grandchild’s sex in our models assessing the impact of grandmother’s smoking in pregnancy since that is unavailable in UK Biobank, which may also reduce our statistical power. MR studies with larger sample sizes and hence greater statistical power are needed to further investigate transgenerational effects of smoking heaviness, together with studies in which both maternal and offspring genotype are known.

We demonstrated how offspring genotype can be used to proxy for maternal genotype, to investigate causal effects of maternal smoking heaviness in pregnancy when maternal genotype is unavailable. We demonstrated our proxy G× E approach by replicating the previously identified effect of heavier smoking on lower offspring birthweight. We found little evidence of a causal effect of maternal smoking heaviness on offspring’s later life outcomes. Finally, we found evidence that the effect of grandmother’s smoking in pregnancy on grandchild’s birthweight may be modulated by mother’s smoking status in pregnancy. Further studies with larger sample sizes are needed to improve statistical power. Maternal drinking heaviness in pregnancy could be another trait to which to apply proxy G × E MR. More broadly in the field of using MR to investigate transgenerational effects, structural equation modelling could be used to distinguish maternal genetic effects from offspring effects, where either the maternal or offspring genotype is unobserved and modelled as a latent variable.^55–57^

## Supplementary Material

dyz250_Supplementary_Data
