# Supplementary material for: Proxy gene-by-environment Mendelian randomization study confirms a causal effect of maternal smoking on offspring birthweight, but little evidence of long-term influences on offspring health
Source: Int J Epidemiol. 2019 Dec 13;49(4):1207–18. doi: 10.1093/ije/dyz250 (PMC7660158; doi:10.1093/ije/dyz250)
Supplement: dyz250_Supplementary_Data [file dyz250_supplementary_data.pdf]

Proxy gene-by-environment Mendelian randomization study confirms causal effect of maternal smoking on offspring birthweight, but little evidence of long-term influences on offspring health

Qian Yang, Louise A. C. Millard, George Davey Smith

SUPPLEMENTARY MATERIAL

Supplementary Methods

Supplementary Figure 1. Participant flow diagram

Supplementary Figure 2. Illustration of edge cases when deriving participants smoking in pregnancy

Supplementary Figure 3. Directed acyclic graphs (DAGs) illustrating potential collider bias

Supplementary Figure 4. The associations of G1 rs16969968 with G1 height and age at menarche in sensitivity analyses

Supplementary Figure 5. Comparison of statistical power between gene-by-environment (G×E) Mendelian randomization (MR) and proxy G×E MR

Supplementary Figure 6. Data generation mechanism for the simulation

Supplementary Table 1. Characteristics of the UK Biobank participants (G1) by their sex

Supplementary Table 2. Associations of G1 rs16969968 with potential confounders by strata of smoking status in participants (G1) and their mothers (G0), adjusted for the first ten principal components

Supplementary Table 3. Differences in the associations of rs16969968 with 12 outcomes in participants (G1) across maternal (G0) smoking status in pregnancy

Supplementary Table 4. Observational associations of participants mothers' (G0) smoking in pregnancy with participants' (G1) smoking status and all outcomes

Supplementary Table 5. UK Biobank data field of variables used in this study

SUPPLEMENTARY METHODS

Potential confounders in G1

The age of participants at baseline was derived by the UK Biobank based on their date of birth and date of attending the initial assessment. We used Townsend deprivation index and household income as measures of socio-economic position at baseline. Townsend deprivation index is a score measuring material deprivation in the area where participants were living, calculated by UK Biobank using participants' postcode. Participants (except those who living in sheltered accommodation or a care home) were asked to report their average total household income before tax, with five categories ranging from less than £18 000 to greater than £100 000. Sex of participants was acquired from the registry and updated by the participants.

Supplementary Table 5 summarised the UK Biobank fields we used to derive our smoking phenotypes, outcomes, and confounders.

Simulation

We performed two simulations to compare statistical power of proxy G×E MR to that of G×E MR (where maternal [G0] genotype is available).

Simulation A is for offspring (G1) early life outcomes (e.g. birthweight) that should not be affected by G1 smoking, and thus we will not stratify on G1 smoking status. We generated simulated data according to the directed acyclic graphs shown in Supplementary Figure 6A, using the following steps:

(1) We generated G0 rs16969968 according to the allelic dosage distribution in UK Biobank.

(2) We generated G1 rs16969968 according to both the allelic dosage distribution in UK Biobank and G0 rs16969968. For example, mothers with dosage 1 could have offspring with dosage 0, 1 or 2, with probabilities (1 - effect allele frequency [EAF])/2, 1/2 and EAF/2, respectively. The proportions of G0 and G1 in each dosage were shown in Supplementary Figure 6B.

(3) We generated G0 smoking status in pregnancy according to its proportion of smokers and non-smokers in UK Biobank.

(4) Among smokers, we generated G0 smoking heaviness as a linear effect of G0 rs16969968 (1 cigarette/day/allele[(1)]) and log-normal distributed random error (as observed in UK Biobank).

(5) We generated a standardised continuous outcome in the offspring (G1). Among G0 non-smokers this was generated randomly from a normal distribution. Among G0 smokers, we generated the outcome as a linear function of G0 smoking heaviness and normally distributed random error (as was used for non-smokers).

We repeated our simulation A with different effect sizes of G0 smoking heaviness (i.e. 0.01, 0.025, 0.05, 0.075 and 0.1 SD/cigarette) for step (5) and different total sample sizes (i.e. 100 000, 500 000, 1 000 000 and 5 000 000). Given an effect size and a sample size, we simulated 1000 times. In each time, we stratified on G0 smoking status, estimated G0 rs16969968 – G1 outcome associations for G×E MR and G1 rs16969968 – G1 outcome associations for proxy G×E MR in each stratum, and then identified the strength of interaction between G0 strata using Cochran's Q test for heterogeneity.

Simulation B is for G1 later life outcomes (e.g. adulthood body mass index [BMI]) that would be affected by G1 smoking, and thus we will further stratify on G1 smoking status. We generated

simulated data in the same way as steps (1-4). We also generated G1 smoking status and G1 smoking heaviness following the same rules in steps (3-4). Finally, we generated a standardised continuous outcome in G1. Among G1 non-smokers, this was generated in the same way as step (5). Among G1 smokers, we further include a linear effect of G1 smoking heaviness (0.1 SD/cigarette). We repeated our simulation B in the same way as A. In each time, we stratified on both G0 and G1 smoking status, estimated those genetic associations in A, and then within each G1 stratum identified the strength of interaction between G0 strata (e.g. G0 smokers & G1 smokers versus G0 non-smokers & G1 smokers).

Given an effect size and a sample size, the statistical power would be the times (when the interaction P-value is smaller than 0.05) out of 1000. We plotted the power in Supplementary Figure 5. Simulation code is provided in the GitHub repository [https://github.com/MRCIEU/MR-maternal-smoking/tree/master/power_simulation].

SUPPLEMENTARY FIGURES

Supplementary Figure 1. Participant flow diagram

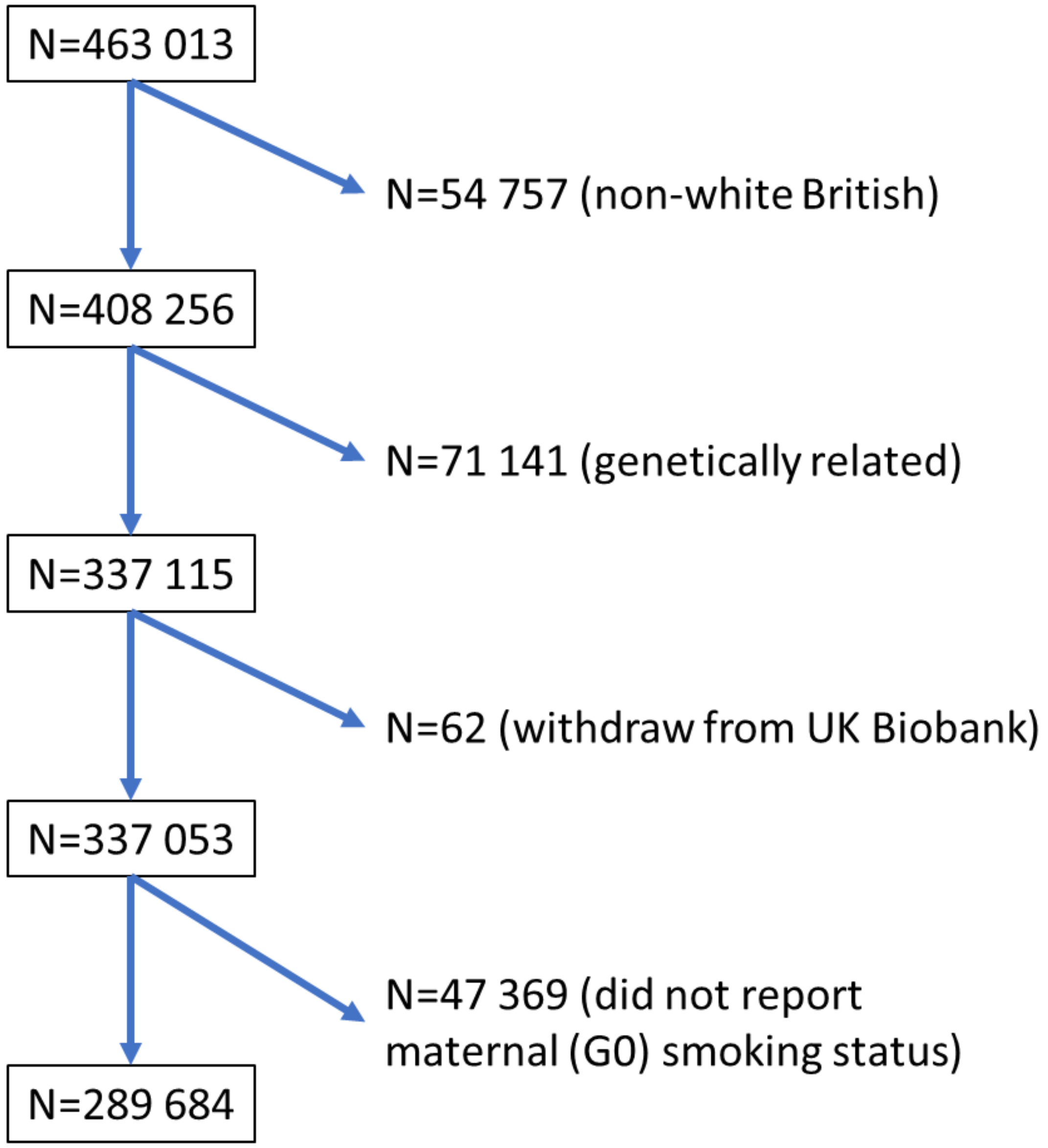

Note: The 463,013 participants are of European descent, have genetic sex same as reported sex, either XX or XY in sex chromosome, and no outliers in heterozygosity and missing rates.[(2)]

Supplementary Figure 2. Illustration of edge cases when deriving participants smoking in pregnancy

A) Participants started smoking in the same year as the first live birth (N=538)

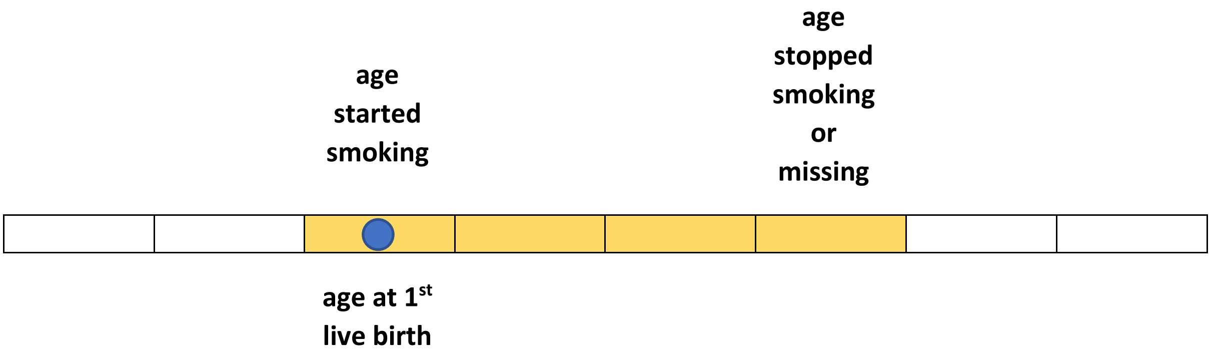

B) Participants stopped smoking in the same year as the first live birth (N=1129)

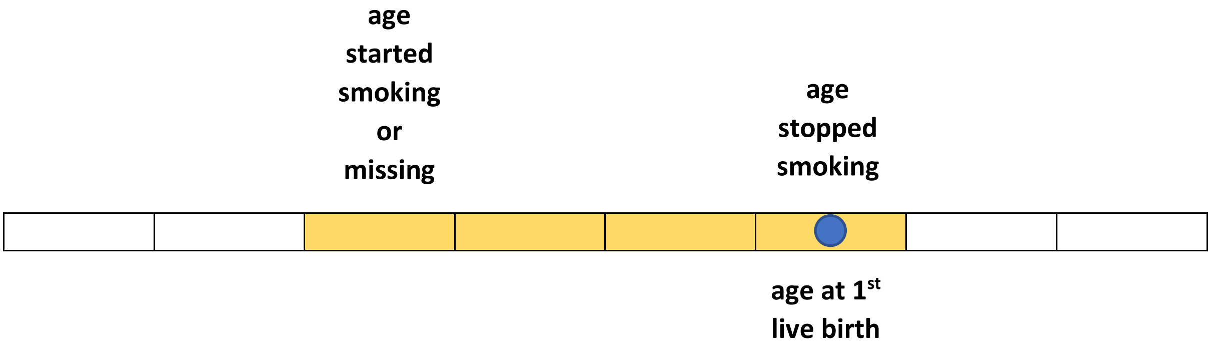

C) Participants stopped smoking in the year before the first live birth (N=1017)

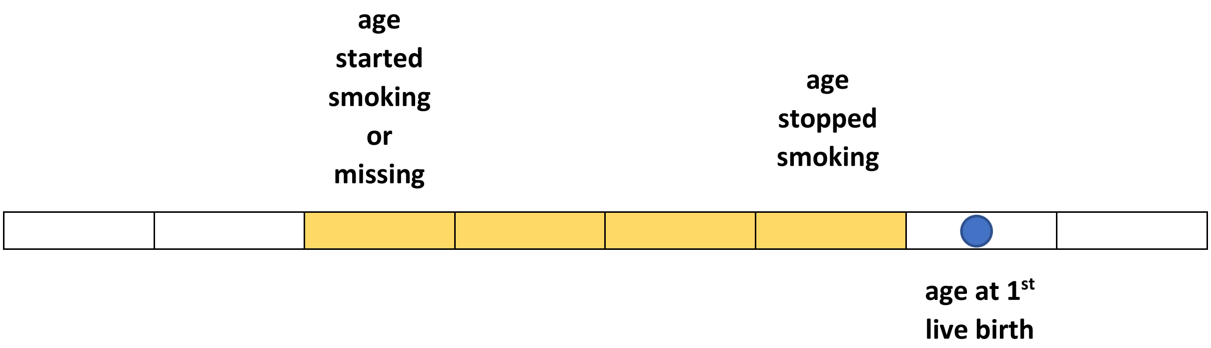

This figure illustrates our derivation of female participants smoking status in their pregnancy resulting in their first live birth. For example, a participant reporting her first live birth occurring at 25 years old could have been pregnant at 25 years old only, or both 24 and 25 years old. If she was a current smoker and started smoking at 25 years old, it was unknown if this was after or during her pregnancy (Supplementary Figure 2A). If she was a current smoker and started smoking at 24 years old, she smoked at least in her late pregnancy. Similarly, if she stopped smoking at 24 or 25 years old (Supplementary Figure 2B&C), it was unknown if this was before or during their pregnancy. If she stopped smoking at 23 years old, she did not smoke during her pregnancy.

Supplementary Figure 3. Directed acyclic graphs (DAGs) illustrating potential collider bias

(A) G×E MR stratifying on maternal (G0) smoking status in pregnancy

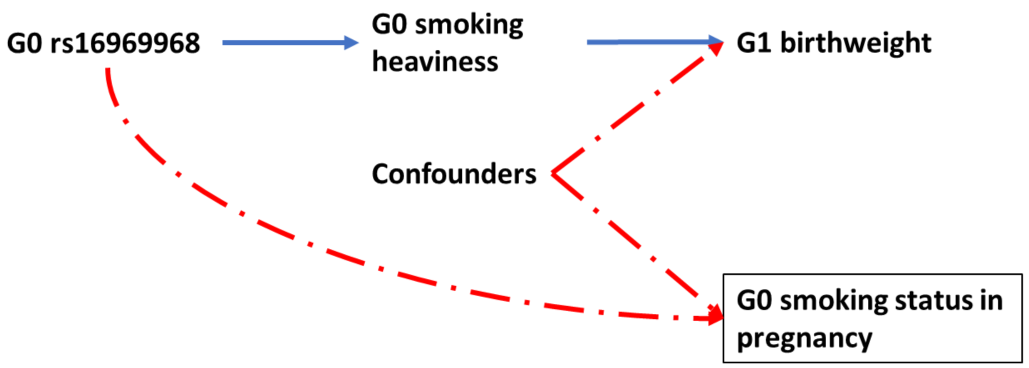

(B) G×E MR stratifying on both maternal (G0) smoking status during pregnancy and offspring (G1) smoking status

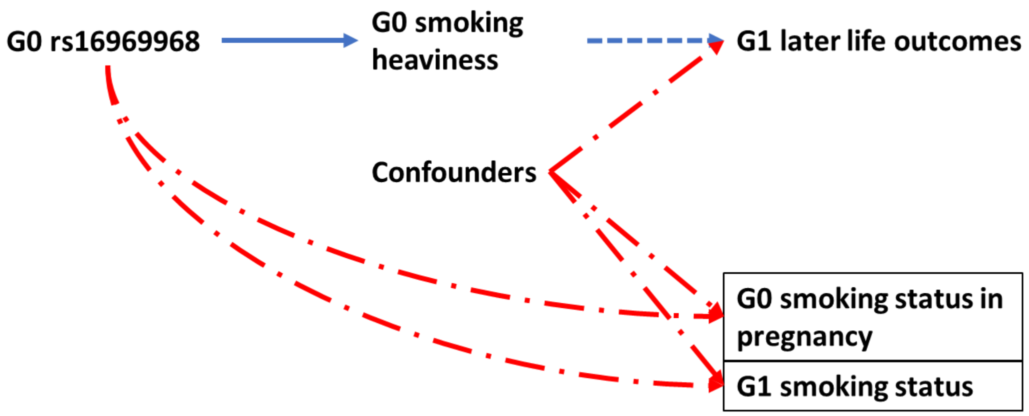

(C) G×E MR testing the effect of grandmother (G0) smoking on grandchild (G2) birthweight, by stratifying on both G0 smoking status in pregnancy and G1 smoking status in pregnancy

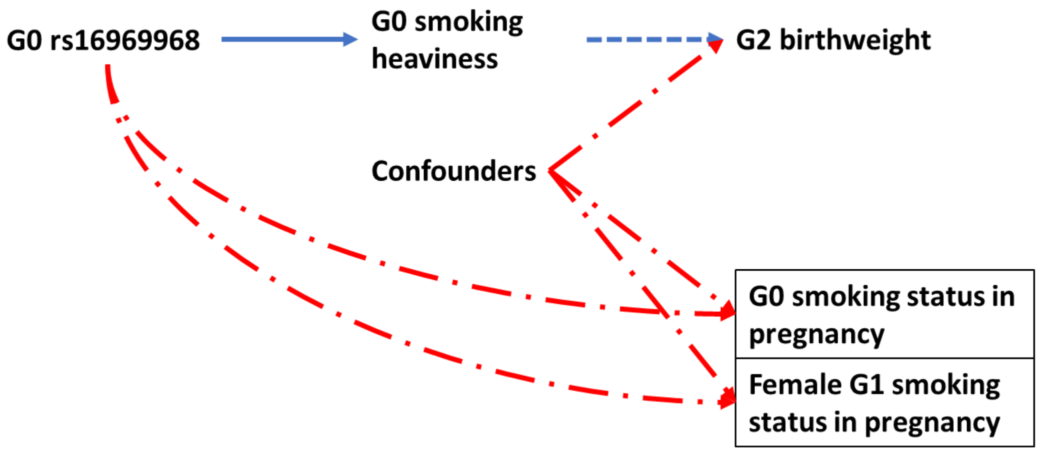

These DAGs show example alternative pathways that may bias associations between the variant and the outcome, due to conditioning on a collider.

Generation (G)0: UK Biobank participants' mother; G1: UK Biobank participants themselves; G2: First child of UK Biobank women participants.

Boxes around a phenotype denote this phenotype is being conditioned upon. Blue solid arrow denotes known association, blue dashed arrow denotes the hypothesis we are testing, and red dashed-dotted arrows denote that conditioning on the collider induced an association between parents of the collider – i.e. an association between genotype and the confounders.

(A) The smoking heaviness variant (rs16969968) has been shown to influence smoking status in pregnancy.[3] In this DAG, G1 birthweight is related to G0 smoking status in pregnancy because they have a common cause (e.g. G0 socioeconomic status [SES]). Therefore, G0 smoking status in pregnancy would be a collider such that conditioning on it induces an alternative pathway between rs16969968 and G1 birthweight that is not via smoking heaviness (shown as — · →). This may bias the association of rs16969968 with G1 birthweight. For example, G0 individuals with more smoking-increasing alleles would be more likely to smoke in pregnancy. G0 individuals with lower SES would be more likely to smoke in pregnancy and concurrently have a lower G1 birthweight. The observed association of G0 rs16969968 with G1 birthweight would include not only the true adverse effect via G0 smoking heaviness but also an adverse effect via conditioning on G0 smoking status in pregnancy, and thus bias the true estimate away from the null.

(B) Besides the potential collider bias described in (A), our proxy (i.e. G1 rs16969968) is weakly associated with G1 smoking status. In this DAG, G1 later life outcomes are also related to G1 smoking status via a common cause (e.g. G1 SES). Therefore, G1 smoking status would be a collider such that conditioning on it induces an alternative pathway between rs16969968 with G1 later life outcomes that is not via smoking heaviness (shown as — · →). This may bias the association of rs16969968 with G1 later life outcomes.

(C) Besides the potential collider bias described in (A), our proxy (i.e. G1 rs16969968) is associated with female G1 smoking status in pregnancy. In this DAG, G2 birthweight is related to female G1 smoking status in pregnancy because they have common causes (e.g. G1 SES). Therefore, female G1 smoking status in pregnancy would be a collider such that conditioning on it induces an alternative pathway between rs16969968 with G2 birthweight that is not via smoking heaviness (shown as — · →). This may bias the association of rs16969968 with G2 birthweight.

Other sources of bias regarding conditioning on a downstream effect (e.g. missingness) of exposures or outcomes have been discussed by Hughes et al.[4]

Supplementary Figure 4. The associations of G1 rs16969968 with G1 height and age at menarche in sensitivity analyses

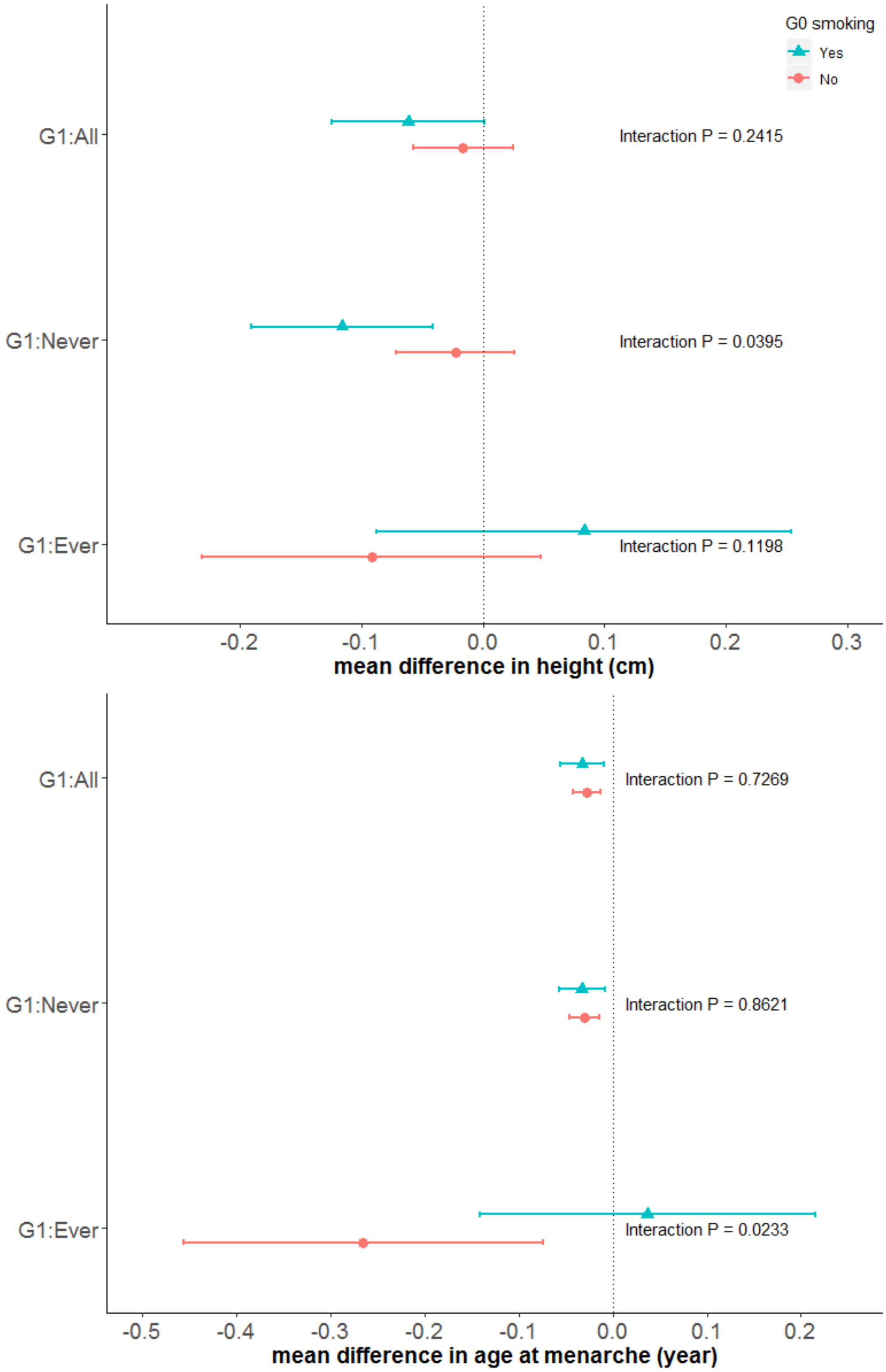

Generation (G)0: UK Biobank participants' mother; G1: UK Biobank participants themselves. Estimates are the mean difference of G1 outcome per each smoking-heaviness increasing allele of rs16969968. G1 were grouped according to whether they were ever smokers before achieving their adulthood height or their age at menarche. G1 who started smoking at the same age of achieving their adulthood height or at menarche were removed from analyses due to uncertainty.

Supplementary Figure 5. Comparison of statistical power between gene-by-environment (G×E) Mendelian randomization (MR) and proxy G×E MR

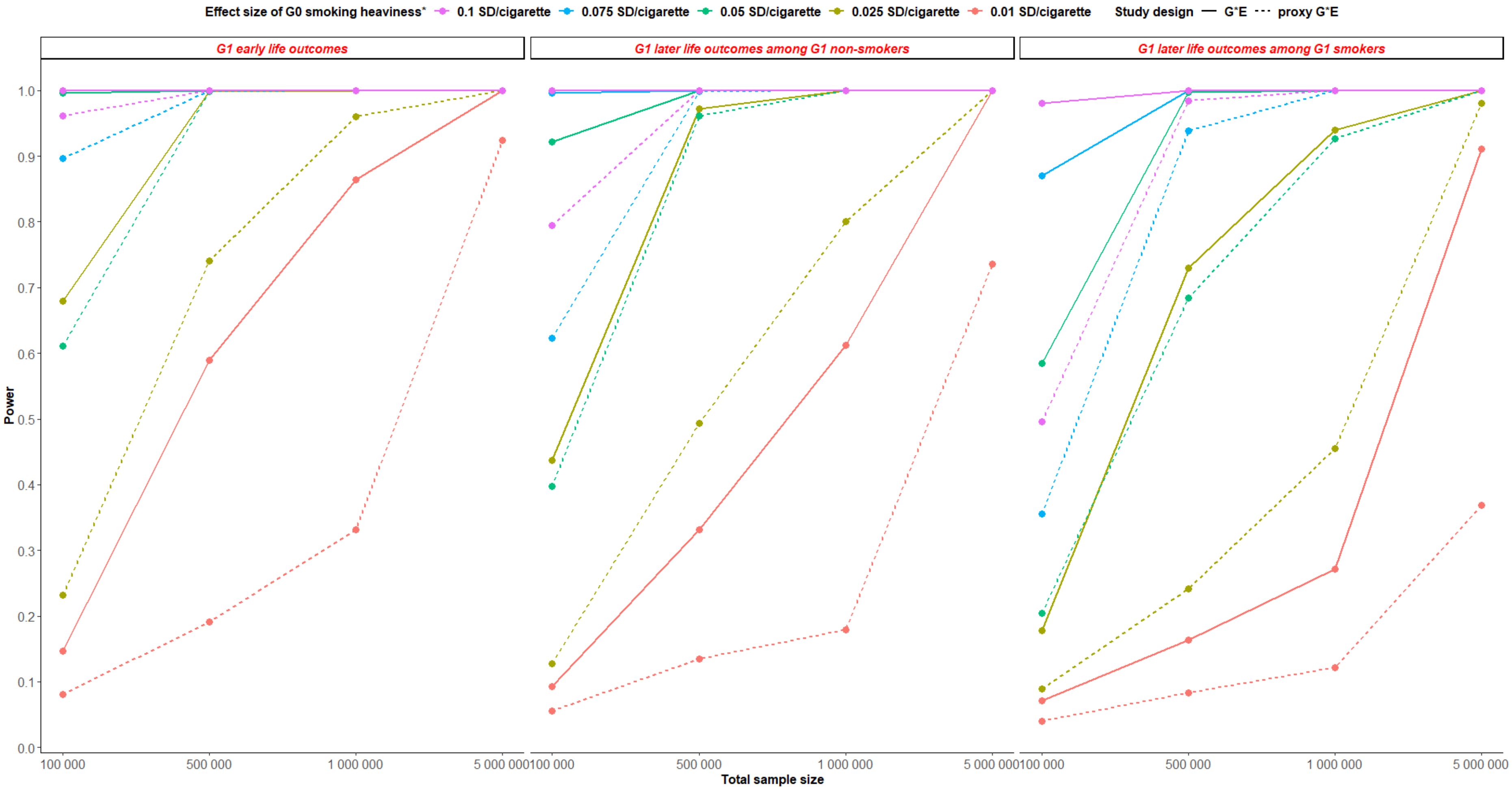

*For example, if the outcome is G1 age at menarche and its SD is 1.6 years in UK Biobank, 0.01 SD/cigarette will be equivalent to 5.84 days/cigarette.

Supplementary Figure 6. Data generation mechanism for the simulation

(A)

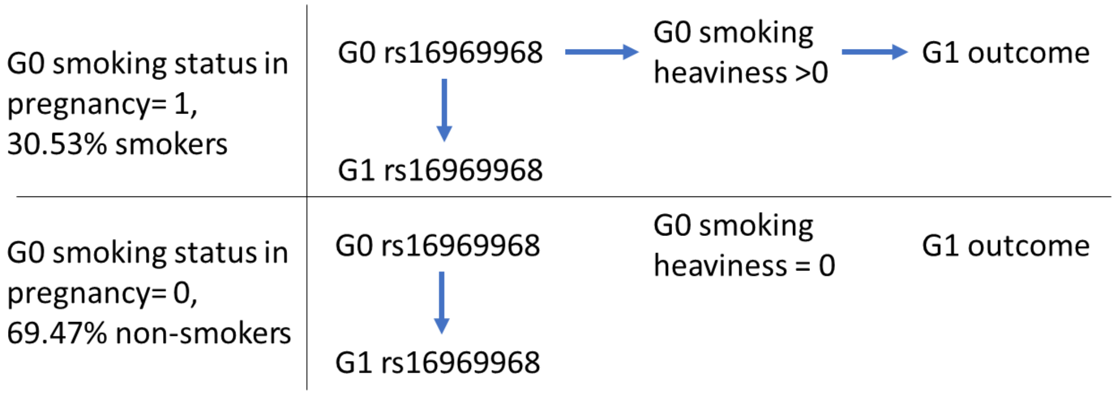

(B)

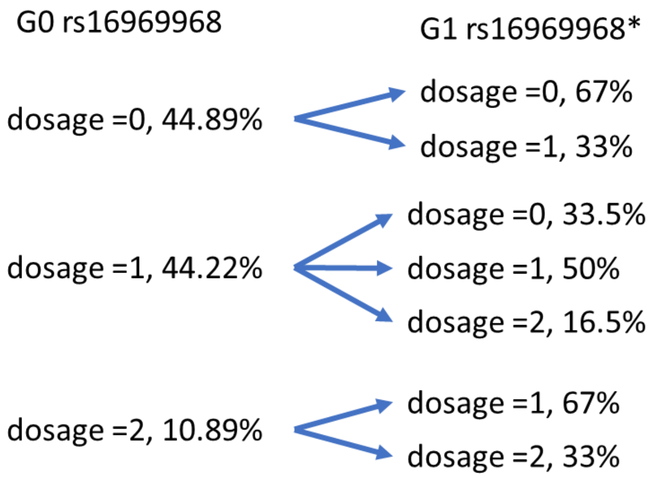

*The effect allele frequency is 33% in UK Biobank participants, and we assume this frequency remains the same in their mothers and fathers.

SUPPLEMENTARY TABLES

Supplementary Table 1. Characteristics of the UK Biobank participants (G1) by their sex

Variable		Total N	Sex[1]	
			Men	Women
Smoking				
Maternal (G0) smoking in pregnancy	*Yes*	88 447	41 793 (31.5)	46 656 (29.7)
	No	201 237	90 889 (68.5)	110 348 (70.3)
Smoking status of participants (G1)	*Current*	27 420	14 924 (11.3)	12 496 (8.0)
	Former	99 932	51 209 (38.7)	48 723 (31.1)
	Never	161 398	66 108 (50.0)	95 290 (60.9)
Participants (G1) smoking in pregnancy[2]	*Yes*	19 061	Not applicable	19 061 (19.1)
	No	80 552		80 552 (80.9)
Outcome in participants (G1)				
Birthweight (kg)		171 784	3.45 ± 0.68	3.24 ± 0.63
Standing height (cm)		289 050	176.04 ± 6.75	162.77 ± 6.22
Body mass index (kg/m^2)		288 775	27.79 ± 4.21	26.98 ± 5.12
Forced vital capacity (L)		218 378	4.48 ± 0.87	3.21 ± 0.63
Forced expiratory volume in 1-second (L)		218 378	3.37 ± 0.73	2.44 ± 0.52
Asthma	*Case*	30 751	13 008 (9.8)	17 743 (14.6)
	Control[3]	258 639	119 531 (90.2)	139 108 (88.7)
Age at menarche (year)		152 991	Not applicable	12.95 ± 1.60
Systolic blood pressure (mmHg)		289 430	141.15 ± 17.44	135.33 ± 19.13
Diastolic blood pressure (mmHg)		289 431	84.14 ± 10.00	80.64 ± 9.95
Year of Education (year)		287 198	15.44 ± 5.06	14.63 ± 5.05
Fluid intelligence score		148 462	6.17 ± 2.18	5.95 ± 2.03
Depression/anxiety	*Case*	102 637	36 333 (27.4)	66 304 (42.3)
	Control	186 743	96 197 (72.6)	90 546 (57.7)
Happiness	*Extremely happy*	5417	2727 (6.2)	2690 (5.2)
	Very happy	37 872	17 670 (40.1)	20 202 (39.2)
	Moderately happy	48 223	21 618 (49.0)	26 605 (51.6)
	Moderately unhappy	3358	1677 (3.8)	1681 (3.3)
	Very unhappy	566	283 (0.6)	283 (0.5)
	Extremely unhappy	188	108 (0.2)	80 (0.2)
Outcome in the first child of female participants (G2)				
Birthweight (kg)		126 122	Not applicable	3.19 ± 0.55
Potential confounder in participants (G1)				
Age (year)		289 684	56.97 ± 8.12	56.48 ± 7.95
Age at first live birth (years)		107 277	Not applicable	25.48 ± 4.55
Deprivation index		289 334	-1.59 ± 2.96	-1.64 ± 2.86
Household income	*less than £18,000*	52 806	22 438 (18.7)	30 369 (23.1)
	£18,000 to £30,999	63 807	29 168 (24.3)	34 644 (26.4)
	£31,000 to £51,999	66 999	32 887 (27.4)	34 117 (26.0)
	£52,000 to £100,000	53 588	27 878 (23.2)	25 712 (19.6)
	greater than £100,000	14 235	7659 (6.4)	6576 (5.0)

[1] Mean ± standard deviation for continuous variables and N (column %) for categorical variables.

[2] Participants smoking in pregnancy was derived from G1 age at first live birth and the ages they reported starting and stopping smoking. As these ages were recorded as a whole number of years, it was not always possible to determine whether a woman was a smoker in pregnancy (see the Methods section in the main paper and Supplementary Figure 2 for details).

[3] Controls were participants who did not indicate having asthma diagnosed by a doctor.

Supplementary Table 2. Associations of G1 rs16969968 with potential confounders by strata of smoking status in participants (G1) and their mothers (G0), adjusted for the first ten principal components

Confounder	By G1 smoking status	Overall	By G0 smoking status in pregnancy	
			Yes	No
Age (years)[1]	All participants	-0.018 (-0.062, 0.026)	-0.080 (-0.156, -0.004)	0.013 (-0.040, 0.067)
	Current smokers	-0.073 (-0.218, 0.072)	-0.092 (-0.325, 0.141)	-0.061 (-0.245, 0.124)
	Former smokers	-0.076 (-0.149, -0.006)	-0.044 (-0.170, 0.083)	-0.087 (-0.174, -0.001)
	Ever smokers	-0.089 (-0.154, -0.024)	-0.062 (-0.175, 0.051)	-0.097 (-0.176, -0.017)
	Never smokers	0.054 (-0.005, 0.113)	-0.074 (-0.177, 0.028)	0.113 (0.041, 0.185)
	Female participants	0.013 (-0.046, 0.072)	-0.059 (-0.163, 0.045)	0.049 (-0.022, 0.121)
	Female smokers in pregnancy	-0.159 (-0.315, -0.003)	0.037 (-0.215, 0.290)	-0.291 (-0.484, -0.095)
	Female non-smokers in pregnancy	0.055 (-0.026, 0.136)	-0.077 (-0.220, 0.067)	0.121 (0.023, 0.220)
Age at first live birth (years)[1]	Female participants	0.019 (-0.022, 0.060)	0.050 (-0.024, 0.125)	0.015 (-0.034, 0.063)
	Female smokers in pregnancy	0.015 (-0.072, 0.101)	0.010 (-0.128, 0.148)	0.017 (-0.093, 0.127)
	Female non-smokers in pregnancy	0.024 (-0.026, 0.074)	0.068 (-0.026, 0.163)	0.017 (-0.041, 0.076)
Deprivation index[1]	All participants	0.010 (-0.006, 0.026)	0.002 (-0.029, 0.032)	0.011 (-0.007, 0.030)
	Current smokers	0.018 (-0.042, 0.079)	0.019 (-0.084, 0.123)	0.018 (-0.056, 0.093)
	Former smokers	0.008 (-0.019, 0.035)	-0.014 (-0.065, 0.038)	0.015 (-0.017, 0.047)
	Ever smokers	0.016 (-0.010, 0.041)	-0.002 (-0.050, 0.045)	0.022 (-0.008, 0.051)
	Never smokers	0.012 (-0.008, 0.032)	0.014 (-0.024, 0.052)	0.009 (-0.014, 0.032)
	Female participants	-0.002 (-0.023, 0.019)	-0.018 (-0.059, 0.023)	0.002 (-0.022, 0.027)
	Female smokers in pregnancy	-0.026 (-0.092, 0.040)	-0.098 (-0.208, 0.011)	0.021 (-0.062, 0.103)
	Female non-smokers in pregnancy	-0.002 (-0.029, 0.026)	-0.022 (-0.076, 0.031)	0.002 (-0.029, 0.034)
Years of education[1]	All participants	0.036 (0.008, 0.064)	0.045 (-0.006, 0.097)	0.036 (0.003, 0.069)
	Current smokers	0.030 (-0.064, 0.124)	0.092 (-0.065, 0.249)	-0.006 (-0.123, 0.111)
	Former smokers	0.043 (-0.005, 0.091)	0.022 (-0.068, 0.112)	0.055 (-0.003, 0.112)
	Ever smokers	0.038 (-0.006, 0.081)	0.038 (-0.040, 0.117)	0.040 (-0.012, 0.091)

	Never smokers	0.026 (-0.010, 0.062)	0.036 (-0.032, 0.103)	0.026 (-0.017, 0.069)
	Female participants	0.050 (0.012, 0.088)	0.064 (-0.006, 0.133)	0.049 (0.005, 0.094)
	Female smokers in pregnancy	0.035 (-0.074, 0.144)	0.155 (-0.022, 0.332)	-0.038 (-0.176, 0.100)
	Female non-smokers in pregnancy	0.046 (-0.006, 0.098)	0.095 (-0.002, 0.191)	0.033 (-0.029, 0.095)
Household income (compare to less than £18,000)[2]	All participants	0.996 (0.986, 1.007)	0.992 (0.974, 1.011)	0.998 (0.986, 1.011)
	Current smokers	0.979 (0.947, 1.013)	0.980 (0.926, 1.037)	0.978 (0.937, 1.021)
	Former smokers	0.993 (0.975, 1.010)	0.995 (0.963, 1.028)	0.992 (0.971, 1.013)
	Ever smokers	0.988 (0.972, 1.004)	0.990 (0.962, 1.018)	0.987 (0.969, 1.006)
	Never smokers	0.999 (0.985, 1.013)	0.988 (0.963, 1.013)	1.003 (0.987, 1.020)
	Female participants	0.994 (0.980, 1.009)	0.991 (0.965, 1.018)	0.996 (0.979, 1.014)
	Female smokers in pregnancy	1.000 (0.959, 1.043)	1.012 (0.946, 1.082)	0.991 (0.940, 1.046)
	Female non-smokers in pregnancy	1.006 (0.986, 1.027)	1.004 (0.967, 1.042)	1.008 (0.984, 1.033)
Sex (compare to female)[3]	All participants	0.995 (0.984, 1.006)	0.987 (0.967, 1.007)	0.998 (0.985, 1.011)
	Current smokers	0.994 (0.959, 1.030)	0.992 (0.935, 1.052)	0.995 (0.951, 1.041)
	Former smokers	1.004 (0.985, 1.023)	1.005 (0.972, 1.040)	1.003 (0.981, 1.026)
	Ever smokers	1.002 (0.986, 1.019)	1.003 (0.973, 1.033)	1.002 (0.982, 1.023)
	Never smokers	0.993 (0.978, 1.008)	0.977 (0.951, 1.004)	0.999 (0.981, 1.017)

[1] Results were from linear regression. Estimates are mean difference of confounder per each smoking-heaviness increasing allele of rs16969968.

[2] Results were from ordinal logistic regression for household income (less than £18 000 = 1, £18 000 to £30 999 = 2, £31 000 to £51 999 = 3, £52 000 to £100 000 = 4, greater than £100 000 = 5). Estimates are the change in odds of being in a higher level of household income per each smoking-heaviness increasing allele of rs16969968.

[3] Results from logistic regression for sex. Estimates are the change in odds of being male rather than female per each smoking-heaviness increasing allele of rs16969968.

Supplementary Table 3. Differences in the associations of rs16969968 with 12 outcomes in participants (G1) across maternal (G0) smoking status in pregnancy

G1 outcome (associations shown in Figure 2)	By G1 smoking status[1]	Interaction P-value[2]
Height	All participants	0.2415
	Never smokers	0.0291
	Ever smokers	0.5491
Body mass index	All participants	0.8710
	Never smokers	0.4258
	Former smokers	0.2756
	Current smokers	0.7849
Forced expiratory volume in 1-second	All participants	0.4603
	Never smokers	0.6462
	Former smokers	0.4970
	Current smokers	0.7575
Forced vital capacity	All participants	0.6788
	Never smokers	0.3199
	Former smokers	0.9120
	Current smokers	0.1879
Asthma	All participants	0.9756
	Never smokers	0.7751
	Ever smokers	0.8507
Systolic blood pressure	All participants	0.3985
	Never smokers	0.2725
	Former smokers	0.7075
	Current smokers	0.5221
Diastolic blood pressure	All participants	0.5861
	Never smokers	0.9896
	Former smokers	0.1164
	Current smokers	0.2265
Age at menarche	All participants	0.7269
	Never smokers	0.8938
	Ever smokers	0.8250
Year of education	All participants	0.9796
	Never smokers	0.8282
	Ever smokers	0.8791
Fluid intelligence score	All participants	0.9067
	Never smokers	0.2581
	Former smokers	0.1856
	Current smokers	0.8321
Depression/anxiety	All participants	0.8368
	Never smokers	0.8355
	Former smokers	0.9112
	Current smokers	0.6190
Happiness	All participants	0.0668
	Never smokers	0.1529
	Ever smokers	0.2689

[1] We combined current and former smokers into ever smokers for some outcomes given smoking cessation may not influence them rapidly.
[2] Interaction P-value was obtained using Cochran's Q statistic for the heterogeneity in the association of rs16969968 with each outcome between participants whose mothers did versus did not smoke.

Supplementary Table 4. Observational associations of participants mothers' (G0) smoking in pregnancy with participants' (G1) smoking status and all outcomes

Dependent variables	Model 1		Model 2	
Smoking in participants (G1)	*OR (95% CI)*	*P-value*		
Smoking status (ever vs never)	1.073 (1.056, 1.090)	3.7×10^{-18}	Not applicable	
Smoking in pregnancy (smoker vs non-smoker)	1.494 (1.446, 1.544)	1.3×10^{-126}	Not applicable	
Outcome in participants (G1)	*Mean difference (95% CI)*	*P-value*	*Mean difference (95% CI)*	*P-value*
Birthweight (kg)[1]	-0.104 (-0.111, -0.097)	6.6×10^{-198}	-0.101 (-0.107, -0.094)	1.1×10^{-182}
Standing height (cm)[1]	-0.509 (-0.582, -0.437)	8.2×10^{-43}	-0.273 (-0.345, -0.200)	1.6×10^{-13}
Body mass index (kg/m^2)[1]	0.850 (0.813, 0.888)	~0	0.732 (0.695, 0.770)	1.9×10^{-319}
Forced vital capacity (L)[1]	-0.037 (-0.045, -0.028)	4.7×10^{-17}	-0.010 (-0.018, -0.001)	2.3×10^{-2}
Forced expiratory volume in 1-second (L)[1]	-0.038 (-0.044, -0.031)	4.0×10^{-29}	-0.015 (-0.022, -0.008)	7.4×10^{-6}
	OR (95% CI)	*P-value*	*OR (95% CI)*	*P-value*
Asthma (case vs control)[1]	1.073 (1.047, 1.101)	4.2×10^{-8}	1.075 (1.048, 1.103)	3.5×10^{-8}
	Mean difference (95% CI)	*P-value*	*Mean difference (95% CI)*	*P-value*
Age at menarche (year)[1]	-0.056 (-0.074, -0.039)	3.5×10^{-10}	-0.070 (-0.087, -0.052)	1.3×10^{-14}
Systolic blood pressure (mmHg)[1]	0.517 (0.377, 0.656)	3.7×10^{-13}	0.436 (0.296, 0.577)	1.2×10^{-9}
Diastolic blood pressure (mmHg)[1]	0.413 (0.333, 0.493)	5.4×10^{-24}	0.393 (0.312, 0.474)	1.9×10^{-21}
Year of Education[1]	-0.796 (-0.836, -0.757)	~0	Not applicable	
Fluid intelligence score[1]	-0.236 (-0.259, -0.212)	1.8×10^{-87}	-0.132 (-0.154, -0.110)	2.0×10^{-31}
	OR (95% CI)	*P-value*	*OR (95% CI)*	*P-value*
Depression/anxiety (case vs control)[1]	1.191 (1.172, 1.211)	4.9×10^{-97}	1.160 (1.141, 1.179)	5.3×10^{-68}
Happiness (extremely happy to extremely unhappy)[1]	0.940 (0.915, 0.965)	4.8×10^{-6}	0.958 (0.932, 0.984)	1.7×10^{-3}
Outcome in the first child of female participants (G2)	*Mean difference (95% CI)*	*P-value*	*Mean difference (95% CI)*	*P-value*
Birthweight (kg)[2]	-0.003 (-0.009, 0.004)	0.44	0.013 (0.005, 0.020)	6.2×10^{-4}

[1] Model 1 adjusted for G1 age; Model 2 adjusted for G1 age, smoking status, education and Townsend deprivation index.

[2] Model 1 adjusted for G1 age; Model 2 adjusted for G1 age, smoking status in pregnancy, education and Townsend deprivation index.

Supplementary Table 6. UK Biobank data field of variables used in this study

Variable	UK Biobank data field
Smoking phenotypes	
Maternal smoking around birth	1787
Smoking status	20116
Age started smoking in current smokers	3436
Age started smoking in former smokers	2867
Age stopped smoking	2897
Outcomes	
Birth weight	20022
Standing height	50
Body mass index (BMI)	21001
Forced expiratory volume in 1-second (FEV1), Best measure	20150
Forced vital capacity (FVC), Best measure	20151
Blood clot, DVT, bronchitis, emphysema, asthma, rhinitis, eczema, allergy diagnosed by doctor	6152
Systolic blood pressure, automated reading	4080
Systolic blood pressure, manual reading	93
Diastolic blood pressure, automated reading	4079
Diastolic blood pressure, manual reading	94
Age when periods started (menarche)	2714
Qualifications	6138
Fluid intelligence score	20016, 20191
Seen doctor (GP) for nerves, anxiety, tension or depression	2090
Seen a psychiatrist for nerves, anxiety, tension or depression	2100
Diagnoses - main ICD10	41202
Diagnoses - secondary ICD10	41204
Happiness	4526
Birth weight of first child	2744
Potential confounder	
Age at recruitment	21022
Age at first live birth	2754
Sex	31
Townsend deprivation index at recruitment	189
Average total household income before tax	738

Further information on these phenotypes can be found by searching the UK Biobank data showcase: http://biobank.ctsu.ox.ac.uk/showcase/search.cgi.

References

1. Munafo MR, Timofeeva MN, Morris RW*, et al.* Association between genetic variants on chromosome 15q25 locus and objective measures of tobacco exposure. *J Natl Cancer Inst*. 2012;**104**:740-748.
2. Mitchell R, Hemani G, Dudding T*, et al.* UK Biobank Genetic Data: MRC-IEU Quality Control, Version 1. 2017. https://data.bris.ac.uk/data/dataset/3074krb6t2frj29yh2b03x3wxj.
3. Freathy RM, Ring SM, Shields B*, et al.* A common genetic variant in the 15q24 nicotinic acetylcholine receptor gene cluster (CHRNA5-CHRNA3-CHRNB4) is associated with a reduced ability of women to quit smoking in pregnancy. *Hum Mol Genet*. 2009;**18**:2922-2927.
4. Hughes RA, Davies NM, Davey Smith G*, et al.* Selection Bias When Estimating Average Treatment Effects Using One-sample Instrumental Variable Analysis. *Epidemiology*. 2019;**30**:350-357.
